# Prophylactic saline-immersion snare-tip vessel coagulation after colorectal endoscopic resection

**DOI:** 10.1055/a-2419-2195

**Published:** 2024-11-28

**Authors:** Antonio Capogreco, Roberto de Sire, Davide Massimi, Ludovico Alfarone, Roberta Maselli, Cesare Hassan, Alessandro Repici

**Affiliations:** 1 9268 Digestive Endoscopy Unit, IRCCS Humanitas Research Hospital, Rozzano, Italy; 2 Gastroenterology, IBD Unit, Department of Clinical Medicine and Surgery, University Federico II, Napoli, Italy; 3 Gastroenterology, Endoscopy Unit, IRCCS Humanitas Research Hospital, Rozzano, Italy; 4 9268 Digestive Endoscopy Unit, IRCCS Humanitas Research Hospital, Rozzano, Italy; 5 9268 Digestive Endoscopy Unit, IRCCS Humanitas Research Hospital, Rozzano, Italy; 6 9268 Digestive Endoscopy Unit, IRCCS Humanitas Research Hospital, Rozzano, Italy; 7 437807 Department of Biomedical Sciences, Humanitas University, Pieve Emanuele, Italy; 8 437807 Department of Biomedical Sciences, Humanitas University, Pieve Emanuele, Italy; 9 9268 Digestive Endoscopy Unit, IRCCS Humanitas Research Hospital, Rozzano, Italy; 10 437807 Department of Biomedical Sciences, Humanitas University, Pieve Emanuele, Italy; 11 9268 Digestive Endoscopy Unit, IRCCS Humanitas Research Hospital, Rozzano, Italy

Endoscopic mucosal resection (EMR) for large colorectal adenomatous lesions is hampered by a relevant risk of post-EMR delayed bleeding 1 2 . Patients who have proximal lesions and/or are on antithrombotic treatment are at higher risk for such delayed bleeding 3 .

Post-EMR prophylactic vessel coagulation has been previously standardized as the application of a low-voltage current using hemostatic forceps 4 . However, this requires a time-consuming device exchange, as well as an additional cost. In addition, the application of a low-voltage current by a relatively large forceps may result in deep thermal injury.

A recent peroral endoscopic myotomy-based series reported that a high-voltage coagulation current delivered through a dedicated knife in a saline-immersion setting maximizes the coagulation effect, preventing unintentional cutting of the vessel wall 5 .

We present the case of an 84-year-old woman who underwent an underwater piecemeal EMR (Captivator II, 15 mm; Boston Scientific, Marlborough, Massachusetts, USA) for a large (50 mm) right colon laterally spreading tumor granular-type without endoscopic features of submucosal invasive cancer. To prevent delayed bleeding, prophylactic saline-immersion coagulation was performed at the end of the procedure ( Video 1 ).

Prophylactic saline-immersion coagulation for prevention of delayed bleeding after endoscopic mucosal resection for right colonic laterally spreading tumor.Video 1

The snare tip was gently placed in contact with the visible vessels and a high-voltage coagulation current (ForcedCOAG E4.0, ERBE VIO3; ERBE Elektromedizin GmbH, Tübingen, Germany) was delivered. This resulted in progressive presealing of the vessels without any cutting effect ( Fig. 1 ). The patient was discharged 4 hours after the procedure with no relevant post-procedural symptoms. No delayed bleeding or other adverse events were reported up to 30 days after the procedure.

**Fig. 1:**
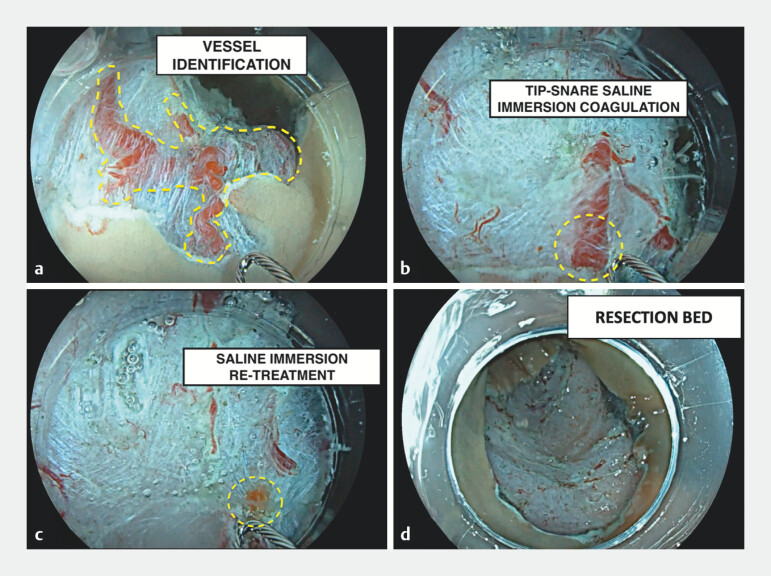
Endoscopic images of saline-immersion coagulation. **a** Blood vessel identification (dashed line) after endoscopic mucosal resection. **b** Prophylactic snare-tip coagulation. **c** The vessels appear whitish after application of the high-current voltage under saline immersion. **d** Resection bed after saline-immersion snare-tip vessel coagulation.

This novel technique aims to reduce the risk of delayed bleeding after endoscopic resection using a one-device, cost-effective, and time-sparing approach. It also highlights the potential applications of saline-immersion coagulation in the field of endoscopy, which appear to be universal and irrespective of the technique, device, or type of current adopted.

Endoscopy_UCTN_Code_TTT_1AQ_2AZ

Citation Format

Endoscopy 2024; 56: E622–E623. doi: 10.1055/a-2353-6039

## Notes

Endoscopy E-Videos https://eref.thieme.de/e-videos
*E-Videos* is an open access online section of the journal *Endoscopy* , reporting on interesting cases and new techniques in gastroenterological endoscopy. All papers include a high-quality video and are published with a Creative Commons CC-BY license. Endoscopy E-Videos qualify for HINARI discounts and waivers and eligibility is automatically checked during the submission process. We grant 100% waivers to articles whose corresponding authors are based in Group A countries and 50% waivers to those who are based in Group B countries as classified by Research4Life (see: https://www.research4life.org/access/eligibility/ ). This section has its own submission website at https://mc.manuscriptcentral.com/e-videos .
